# Bilateral pretibial edema associated with risperidone: A case report

**DOI:** 10.1192/j.eurpsy.2023.2134

**Published:** 2023-07-19

**Authors:** B. Yildiz, F. Alioglu Karayilan, I. Ak

**Affiliations:** 1 Psychiatry, Karabuk University; 2 Psychiatry, Private Praxis, Karabuk, Türkiye

## Abstract

**Introduction:**

Risperidone is a benzisoxazole derivative and is an antipsychotic drug that is frequently used in psychiatric disorders with high binding to 5HT-2A, D2, α1-adrenergic and α2-adrenergic receptors. It is a second generation antipsychotic and the most common side effects are known as extrapyramidal symptoms, weight gain, sedation, hyperprolactinemia, dizziness, insomnia, anxiety and nausea. Edema has been reported in a few cases as a side effect of risperidone in the literature.

**Objectives:**

In this case report, we aimed to present a case of peripheral edema developed in a patient using escitalopram combined with risperidone and to review the literature on this subject.

**Methods:**

A 48-year-old male presented to the outpatient psychiatry clinic with symptoms of difficulty in controlling his irritability and anger, damaging the things around him, depression, loss of interest and desire, sleep disturbance, fatigue, and feelings of worthlessness. The patient also applied to the psychiatry outpatient clinic with similar symptoms 5 months ago, and had been on sertraline 50 mg/day and quetiapine 25 mg/day since then. But it was learned that he did not benefit, so his treatment was adjusted as escitalopram 10 mg/day and risperidone 1 mg/day. The patient, who came to the outpatient clinic control after 1 month and did not describe a similar complaint before, described peripheral edema that developed approximately 2 weeks after he started using psychiatric medication. On examination, 3+ pretibial peripheral edema was detected. Since the patient had a previous history of escitalopram use, it was recommended to continue escitalopram 10 mg/day treatment, and risperidone 1 mg/day was discontinued and he was called for control.

**Results:**

It was observed that the edema completely resolved within 2 weeks following the discontinuation of risperidone treatment.

**Conclusions:**

In conclusion, risperidone is an antipsychotic frequently used in clinical practice, and edema is a rare but important side effect and can be encountered even at low doses. Despite the low incidence, this side effect should be considered by clinicians. There is a need for controlled studies that explain the relationship between risperidone and edema, elucidate the mechanism of edema and investigate its incidence.

**Disclosure of Interest:**

None Declared

